# Networks of blood proteins in the neuroimmunology of schizophrenia

**DOI:** 10.1038/s41398-018-0158-y

**Published:** 2018-06-06

**Authors:** Clark D. Jeffries, Diana O. Perkins, Margot Fournier, Kim Q. Do, Michel Cuenod, Ines Khadimallah, Enrico Domenici, Jean Addington, Carrie E. Bearden, Kristin S. Cadenhead, Tyrone D. Cannon, Barbara A. Cornblatt, Daniel H. Mathalon, Thomas H. McGlashan, Larry J. Seidman, Ming Tsuang, Elaine F. Walker, Scott W. Woods

**Affiliations:** 1 Renaissance Computing Institute, University of North Carolina, Chapel Hill, NC USA; 2 Department of Psychiatry, University of North Carolina, Chapel Hill, NC USA; 3 Department of Psychiatry, Lausanne University Hospital (CHUV), Lausanne, Switzerland; 4 Laboratory of Neurogenomic Biomarkers, Centre for Integrative Biology, and Microsoft Research, Centre for Computational Systems Biology, University of Trento, Trento, Italy; 5 Hotchkiss Brain Institute, Department of Psychiatry, University of Calgary, Calgary, AB Canada; 6 Departments of Psychiatry and Biobehavioral Sciences and Psychology, UCLA, Los Angeles, CA USA; 7 Department of Psychiatry, UCSD, San Diego, CA USA; 8 Department of Psychology, Yale University, New Haven, CT USA; 9 Department of Psychiatry, Zucker Hillside Hospital, Long Island, NY USA; 10 Department of Psychiatry, UCSF and San Francisco VA Healthcare System, San Francisco, CA USA; 11 Department of Psychiatry, Harvard Medical School at Beth Israel Deaconess Medical Center and Massachusetts General Hospital, Boston, MA USA; 12 Department of Psychiatry, Center for Behavioral Genomics UCSD, San Diego, CA USA; 13 Departments of Psychology and Psychiatry, Emory University, Atlanta, GA USA

## Abstract

Levels of certain circulating cytokines and related immune system molecules are consistently altered in schizophrenia and related disorders. In addition to absolute analyte levels, we sought analytes in *correlation networks* that could be prognostic. We analyzed baseline blood plasma samples with a Luminex platform from 72 subjects meeting criteria for a psychosis clinical high-risk syndrome; 32 subjects converted to a diagnosis of psychotic disorder within two years while 40 other subjects did not. Another comparison group included 35 unaffected subjects. Assays of 141 analytes passed early quality control. We then used an unweighted co-expression network analysis to identify highly correlated modules in each group. Overall, there was a striking loss of network complexity going from unaffected subjects to nonconverters and thence to converters (applying standard, graph-theoretic metrics). Graph differences were largely driven by proteins regulating tissue remodeling (e.g. blood-brain barrier). In more detail, certain sets of antithetical proteins were highly correlated in unaffected subjects (e.g. SERPINE1 vs MMP9), as expected in homeostasis. However, for particular protein pairs this trend was reversed in converters (e.g. SERPINE1 vs TIMP1, being synthetical inhibitors of remodeling of extracellular matrix and vasculature). Thus, some correlation signals strongly predict impending conversion to a psychotic disorder and directly suggest pharmaceutical targets.

## Introduction

Circulating levels of immune system proteins and related signaling agents are consistently altered in schizophrenia. This observation includes unmedicated first episode psychosis patients^1,2^ and persons at clinical high-risk who subsequently convert to psychosis^3–5^. Consistent findings include proteins in the immune system acute phase response and in the plasminogen activating system^1^. Many of the proteins influence brain function directly, crossing blood–brain barrier (BBB) and signaling glia or other perivascular cells. These immune signaling molecules also regulate brain function by influencing BBB endothelial cell function and general integrity. Such findings support the hypothesis that psychosis involves brain dysregulation by an altered peripheral immune system and aberrant signaling at BBB.

Persons meeting clinical high-risk criteria have about a 20% risk of developing a psychotic disorder within two years, and that is 100-fold higher than the 0.2% general population risk^6^. Baseline factors differentiating clinical high-risk subjects that convert to psychosis vs do not convert may be of etiologic significance. Previous psychosis risk prediction studies compared levels of immune signaling molecules, individually or in a linear combination^3–5,7^. Since responses of the peripheral immune system are highly coordinated, investigation of the correlation patterns of immune signaling molecules might also be informative. In this analysis we sought networks of highly correlated immune molecules in persons at clinical high-risk who developed psychosis, compared to those who did not develop psychosis over the two-year follow-up period. We also analyzed data from unaffected comparison subjects. In some respects, the correlation networks were strikingly different in converters. Some distinguishing proteins were modulators of extracellular matrix (ECM) components and BBB.

Schizophrenia pervasively impacts brain functions, typically causing in adolescence or early adulthood disability that is chronic and relapsing. Improved clinical outcomes are often associated identification and treatment of the disorder early in its course^8^, implying the desirability of seeking reliable predictors based upon readily accessible biomarkers. Moreover, early indicators might be close to the ultimate causes of schizophrenia.

Thus, researchers (e.g., Domenici et al.^9^ and Dickerson et al.^10^) have investigated in particular proteins and other blood plasma analytes that distinguish unaffected comparison subjects from patients with schizophrenia or subsets of such patients^11,12^. Included among the analytes have been concentrations of circulating cytokines and other immune system signaling molecules. These can be altered in schizophrenia across all stages of the disorder including the prodromal stage.

One key concept of this paper is information from networks. As Fredrickson et al.^13^ stated, “…accumulation of many individually noisy indicator variables can yield highly stable estimates of the underlying factors they share in common.” Thus, we sought networks of highly correlated signals among each of three groups (converters, nonconverters, and unaffected comparison subjects) from our North American Prodrome Longitudinal Study (NAPLS) project^14^. The emphasis was analysis of data collected at the prodrome state (see Supplement Figure S1). To place the present work in context, NAPLS is a multi-site program that has accumulated, cleaned, stored, analyzed, and reported many types of clinical and laboratory assays, leading to proposals of various predictors and mechanisms for the development of psychosis^15^.

Thus, the present emphasis differs from some earlier works in that it is longitudinal and it pertains entirely to networks. The three networks from the three groups are formed by proteins that are highly correlated over subjects in each group, far more highly correlated than could be reasonably explained by chance. All of the reported correlations are positive because there we observed no negative correlations of the same, very high magnitude.

Many of the distinguishing analytes we found turned out to be prominent in the immune system^3^ and its interaction with trophic factors and ECM components. Thus, the proper arena of this paper became neuroimmunology. Particularly in the last decade, many important reports have developed this view of mental illnesses (e.g., Khandaker et al.^16^).

A second key concept is the pleiotropic roles of proteins customarily considered in the context of hemostasis vs hemorrhage. Specifically, as Nave and Ehrenreich demonstrated^17^, “It is becoming apparent that coagulation factors do much more than simply act in the blood-coagulation cascade.” For example, fibrinogen associates with schizophrenia^18^, Alzheimer disease^19^, and multiple sclerosis (MS)^20^.

A third key concept is that the peripheral immune system impacts physiological and pathological brain function^21–23^. We have hypothesized that peripheral immune system dysregulation may contribute to development of psychosis. Furthermore, the construction of the above classifier^3^ survived permutation testing very well, implying that certain peripheral blood plasma proteins and conversion of patients to schizophrenia are associated. Finally, dysregulation of BBB permeability, transendothelial cell migration, or remodeling of brain ECM or vasculature—all impacted by the immune system—could obviously be important in the events leading to frank psychotic mood disorders including schizophrenia.

## Materials and methods

### Subjects

The aims and methods of North American Prodrome Longitudinal Study (NAPLS2) were described in detail previously^24^. Briefly, NAPLS2 is an eight-site observational study of predictors and mechanisms of conversion to psychosis. The NAPLS2 cohort includes 765 subjects at clinical high-risk for psychosis, based on the Criteria of Prodromal States, determined by the Structured Interview for Prodromal Syndromes, and rated with the Scale of Prodromal Symptoms^25^ (see Supplement). In addition, the cohort includes 280 demographically similar unaffected comparison subjects. Subjects were between ages 12 and 35 at baseline. Psychiatric diagnoses were determined by the Structured Clinical Interview for DSM IV^26^. Clinical assessments were performed every six months, and subjects were followed for up to two years. The study was approved by the Institutional Review Board at each site, and each subject provided written informed consent or assent, with a parent or guardian also consenting for minor subjects. Demographics for subjects in the present study are provided in Table 1.

**Table 1:** Demographic and clinical characteristics of study subjects taken at baseline of longitudinal study

	Unaffected comparison (UC) *N* = 35	Clinical high-risk, nonconverters (CHR-NC) *N* = 40	Clinical high-risk, Converters (CHR-C) *N* = 32
Age, average (SD)	20 (4.5)	19.5 (4.6)	19.2 (3.7)
Ancestry			
%Caucasian	60%,	65%	55%
%African	31%	17.5%	21%
%Asian	9%	17.5%	24%
Sex, % female	34%	37.5%	30.3%
SES, average (SD)	4.8 (1.8)	4.5 (2.3)	4.5 (1.8)
Time blood draw, average (SD)	12.:12 pm (1.85 h)	12:39 pm (2.0 h)	11:59 am (1.79 h)
Prescription medication			
Antipsychotic^a^	0%	25%	13%
Antidepressant^b^	1%	30%	25%
Stimulant	0%	8%	6%
Mood stabilizer	0%	5%	3%
Benzodiazepine^c^	0%	5%	13%
NSAID	0%	0%	0%
Antibiotic	0%	0%	0%
Substance Use			
Tobacco use^d^	9%	30%	44%
Alcohol use	46%	48%	38%
Marijuana use^e^	9%	25%	31%
Current co-morbid DSM IV Diagnosis			
Depression^f^	0%	45%	50%
Anxiety Disorders^g,*^	3%	60%	56%

^a^CHR-C vs UC FET *p*-value = 0.047, CHR-NC vs UC Fisher Exact Test (FET) *p*-value = 0.001

^b^CHR-C vs UC FET *p*-value = 0.011, CHR-NC vs UC FET *p*-value = 0.002

^c^CHR-C vs UC FET *p*-value = 0.047

^d^CHR-C vs UC FET *p*-value = 0.001, CHR-NC vs UC FET *p*-value = 0.02

^e^CHR-C vs UC FET *p*-value = 0.020, CHR-NC vs UC FET *p*-value = 0.056

^f^CHR-C vs UC FET *p*-value < 0.0001, CHR-NC vs UC FET *p*-value < 0.0001

^g^CHR-C vs UC FET *p*-value < 0.0001, CHR-NC vs UC FET *p*-value < 0.0001

*Depression disorders include Major Depression, Depressive Disorder Not Otherwise Specified, and Dysthymic Disorder. Anxiety Disorders include Obsessive Compulsive Disorder, Post-Traumatic Stress Disorder, Panic Disorder, Agoraphobia, Social Phobia, Specific Phobia, Generalized Anxiety Disorder

### Plasma analytes

During a baseline visit, blood samples were drawn using Becton Dickenson P100 blood collection tubes containing EDTA (as anticoagulant), proprietary protein stabilizers, and a mechanical separator. Mean processing time to −80 °C storage was 28 min (SD = 2 min). Plasma samples were subsequently sent on dry ice to Myriad RBM (Austin TX), a laboratory that has maintained CLIA Accreditation since 2006. Samples were analyzed with the Human DiscoveryMAP v. 1.0 assays of 185 analytes associated with immune system function, hormonal responses, oxidative stress, and metabolism. In total 141 analytes passed preliminary quality control and were used in our analyses. We standardized to *z*-scores the results for each analyte using the average and SD values of the unaffected comparison subjects. Quality control, normalization methods, and tests with duplicated samples were performed, as previously described^3^.

### Data analyses

We used an unweighted co-expression network analysis to identify highly correlated networks of analytes in each of the three groups. Pearson correlations were calculated using macros and built-in functions in Excel. Limited by the smallest group size (converters, *n* = 32), we performed random re-sampling with replacement 10,000 times by drawing subgroups of 28 subjects from each of the three groups. In other words, the 9870 pairs of analytes were compared in 28-dimensional space 10,000 times to calculate Pearson correlations. We compared those correlation values to a common threshold and then recorded the number of times a pair exceeded the threshold (flowcharts in Supplement Figure S2). The correlation threshold selected was 0.7662; if two 28-dimensional vectors were populated by a Gaussian distribution, then the probability of their correlation exceeding this value would be 1E-6. Thus, with 9870 pairs of analytes, we expect only 1 such value among all pairs about once in 100 trials.

**Fig. 1:**
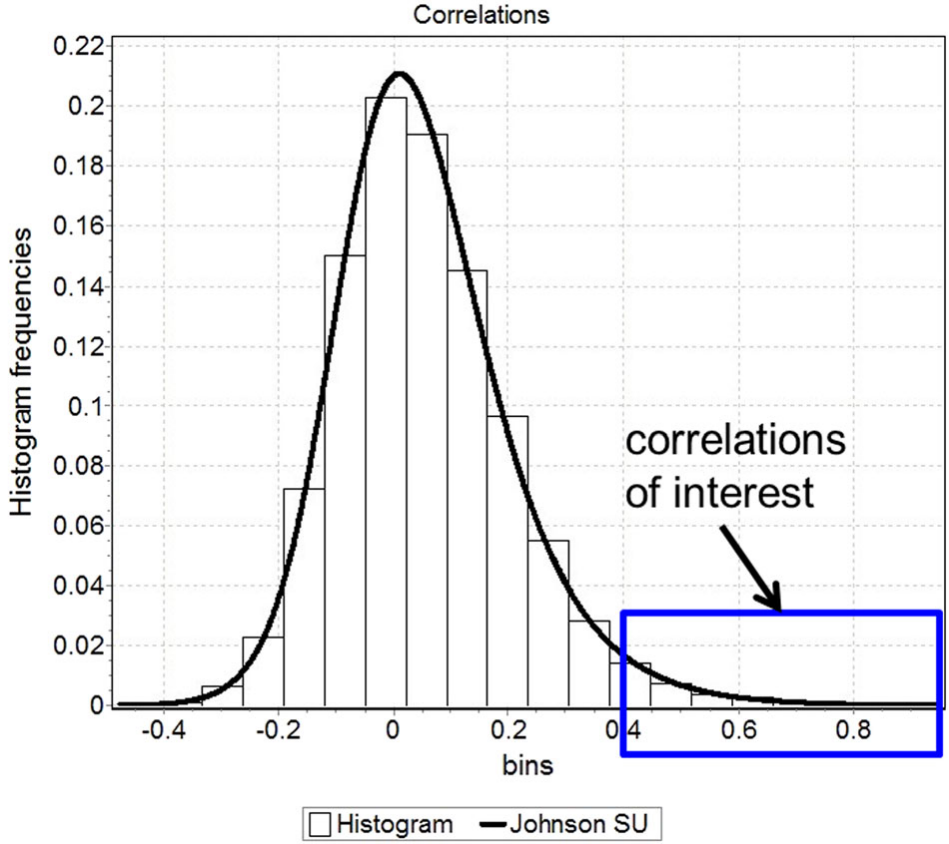
A histogram with 20 bins of 9870 correlation values among 141 analytes over all 107 subjects. The shown distribution is a Johnson S_U_ fit with four parameters: gamma = −1.2967, delta = 2.2624, lambda = 0.26593, xi = −0.12371. The present study is distinguished from many others by focusing on the tail of very strong, positive correlations (blue box) that are not balanced by any negative correlations of the same magnitude

Considering all 107 subjects and all 9870 pairs of analytes at once, distribution analysis using EasyFit (MathWave, Dnepropetrovsk, Ukraine) revealed that no normal distribution fit the observations but the Johnson S_U_ distribution^27^ did. Technically, normal distributions failed to fit the observed distribution of correlations in both Kolmogorov–Smirnov and Anderson–Darling tests for even α = 0.01; by contrast, the Johnson S_U_ distribution achieves α = 0.2 in both tests. The Johnson S_U_ distribution accommodates a one-sided tail of very strong, positive correlations not balanced by even one negative correlation of equal magnitude. Therefore, we focused on only the very strong, positive correlations (Fig. 1).

We validated our approach by first randomly permuting all 107 subjects, then analyzing 28-subject subsets of the pseudo-groups exactly as if they were from the true data. Neither the design nor the implementation of the algorithm conferred any obvious, consistent distinction to edge counts of graphs of very strong correlations in the three pseudo-groups. This result indicates that both the design and implementation of the program correctly achieved unbiased identification of correlation networks.

Measures of network complexity include those of Bonchev and Buck^28^ (see their formulas 19a,b) in which the normalized edge complexity is the number of (undirected) edges *e* divided by the maximum possible number of edges using the same number of vertices *v*, namely, v*(v-1)/2.

Protein network analyses were conducted using Ingenuity Pathway Analysis (IPA) (QIAGEN N.V., Venlo, The Netherlands).

## Results

### Protein correlation networks distinguish the three groups

**Fig. 2:**
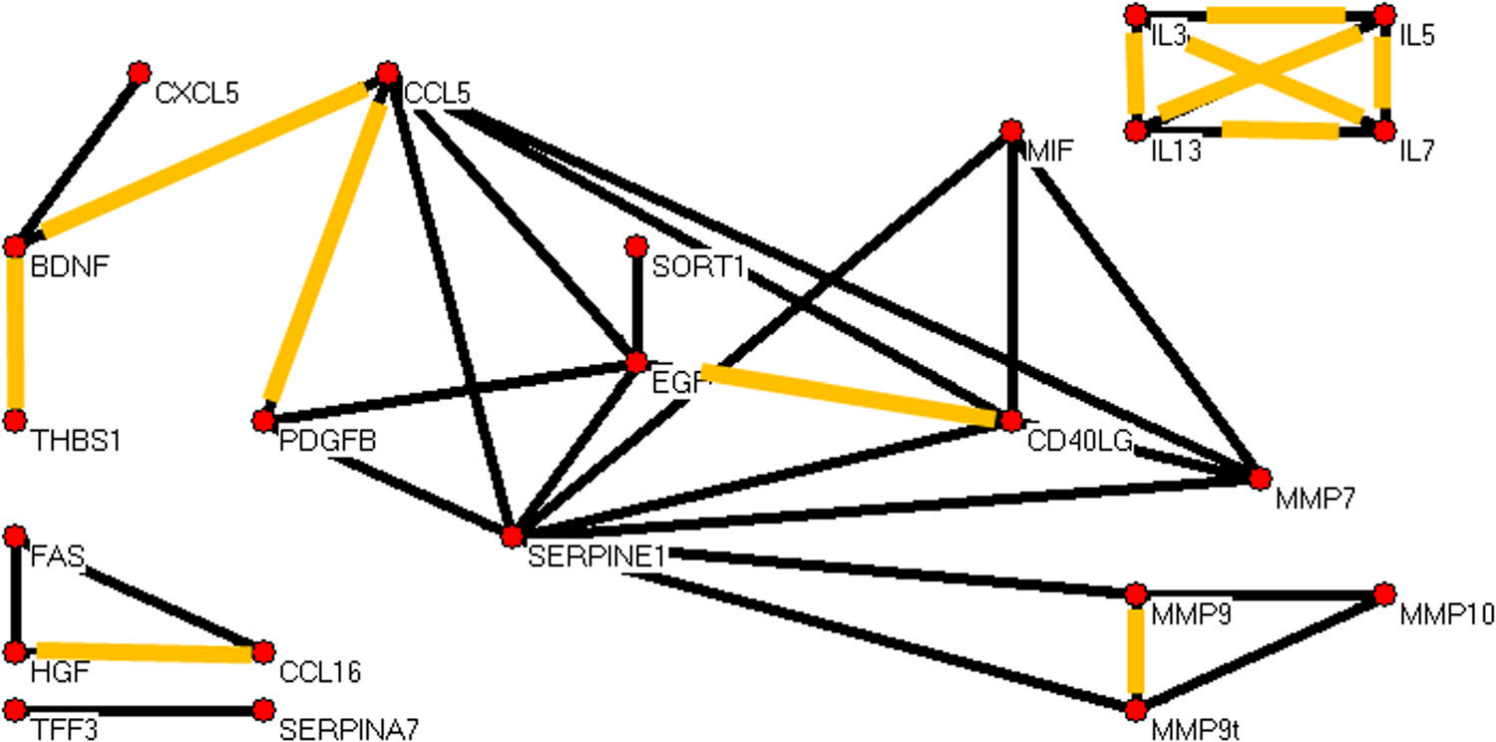
Unaffected comparison subject data yielded a graph of strongly correlated analytes with 23 analytes (vertices) and 34 robust correlations (edges). Blood plasma proteins are labeled by their gene common symbols. The correlations in orange appear in all three graphs. We note eight analytes that include SERPINE1, and in particular SERPINE1 correlations include the matrix metalloproteinases MMP7, MMP9, and MMP10. MMP9t denotes an assay for both pro-MMP9 and mature MMP9

**Fig. 3:**
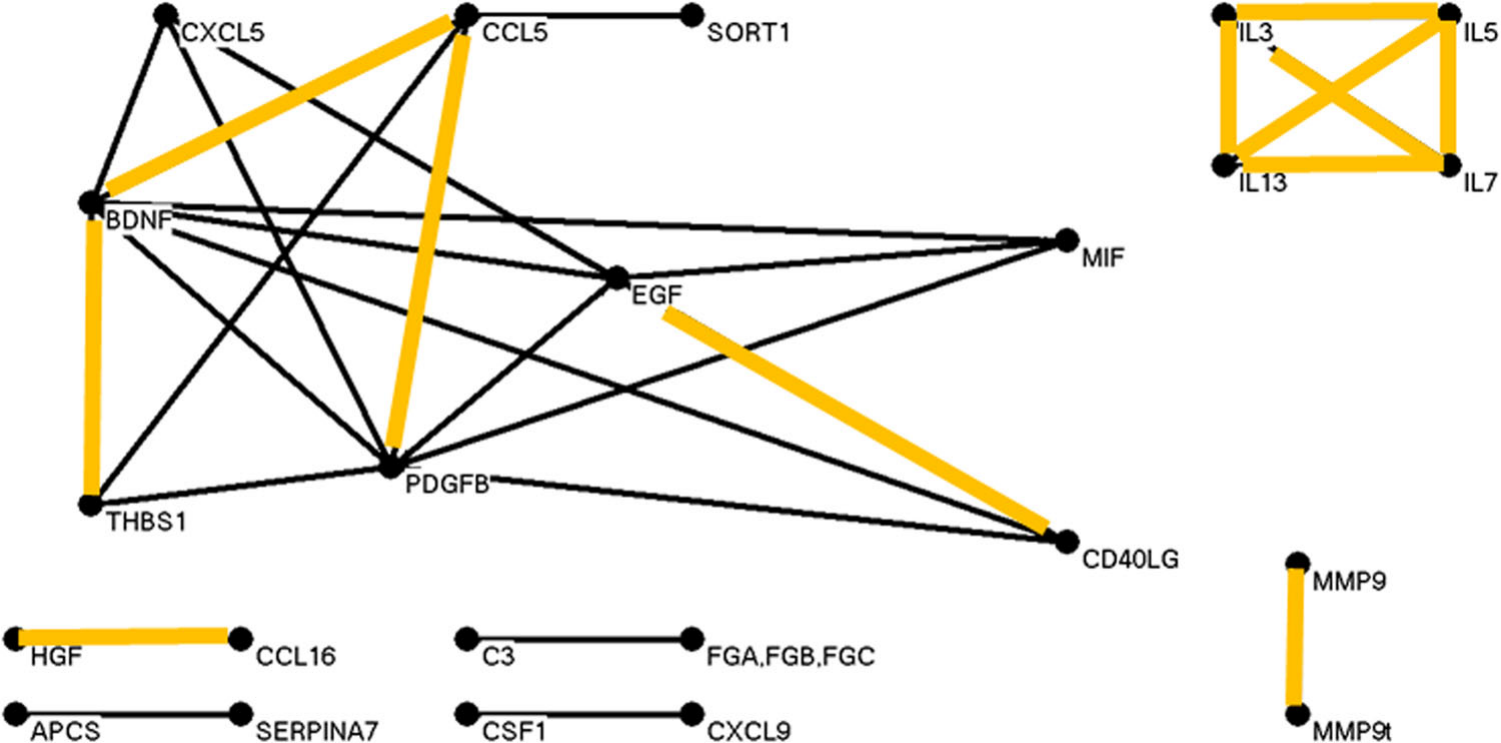
Nonconverter data yielded a graph of strongly correlated analytes with 23 analytes and 30 robust correlations. SERPINE1 correlations are completely absent, suggesting a loss of requlation of expression of the gene

**Fig. 4:**
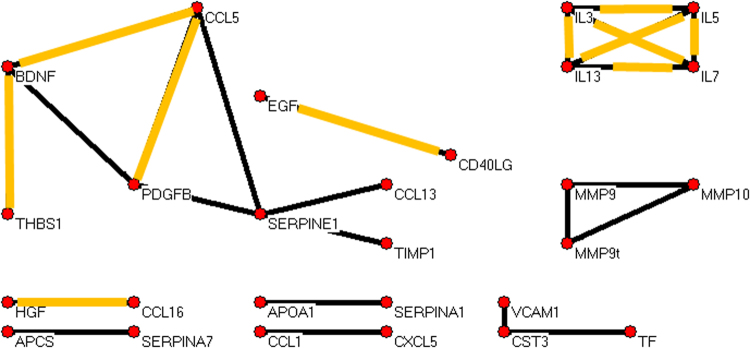
Converter data yielded a graph of strongly correlated analytes with 27 analytes and 24 robust correlations. Four SERPINE1 correlations are present, suggesting changes in requlation of the *SERPINE1* gene compared to unaffected and nonconverter assays. Remarkable is the gained correlation of SERPINE1 and TIMP1 because both proteins inhibit anticogulation and vascular remodeling in some contexts and both generally promote anti-inflammation. Furthermore, TIMP1 is completely absent in unaffected and nonconverter graphs. Also, the strong SERPINE1 correlations with matrix metalloproteinases (MMPs) in the unaffected graph are absent in this converter graph

There was a striking loss of network complexity going from unaffected to nonconverters and thence to converter subjects. Analyte pairs with Pearson correlations exceeding an a priori threshold in at least 5000 of 10,000 random draws of 28 subjects from each group are shown for unaffected subjects (Fig. 2), nonconverters (Fig. 3), and converters (Fig. 4).

For unaffected, nonconverter, and converter groups we calculated a simple metric of graph complexity, namely the ratio of numbers of edges to vertices; respective values were 1.48, 1.30, and 0.89 edges per vertex. Normalized edge complexities were 0.134, 0.119, and 0.062. Moreover, it is visually apparent that the three graphs are quite different in terms of edge densities. Additional graphs and experiments with alternative choices of thresholds led to the same trend and are in the Supplement. This trend toward simplicity going from unaffected to nonconverter to converter for plasma proteins parallels the same for leukocytic microRNAs reported earlier^29^.

Notably, in unaffected subjects there was a network involving the proteins SERPINE1 (plasminogen activator inhibitor-1), MMP7, MMP9 (activated), MMP9t (total), and MMP10 (matrix metalloproteinases). While SERPINE1 is anti-remodeling, the others are pro-remodeling, and a balance (correlation of some pairs) might be expected in hemostasis vs hemorrhage. In contrast, nonconverters and converters lacked these strong SERPINE1 correlations. Furthermore, while the important inhibitor of remodeling TIMP1 (tissue inhibitor of metalloproteinases) was absent (at the chosen threshold for inclusion) from strong correlations in unaffected and nonconverter graphs, it generally exceeded the threshold correlation value with SERPINE1 in converter subjects. In detail, the SERPINE1 vs TIMP1 correlations exceeded the threshold in the three sets of 10,000 random draws of 28 subjects in totals of <10, <10, and >8300 times, respectively. As sets, the actual SERPINE1 and TIMP1 correlation values over 10,000 draws for converter subjects (mean correlation = 0.79, SD = 0.03) tended to be significantly greater (*p*-value < 1E-100) than the values in nonconverters (mean = 0.55, SD = 0.06) and unaffected subjects (mean = 0.50, SD = 0.09). The dramatic distinction of SERPINE1 and TIMP1 correlations in converters is further illustrated in the Supplement (Figure S10).

What could explain the extremely high correlations of SERPINE1 and TIMP1 in converters only? We note that another protein, CTGF (connective tissue growth factor), is known to promote mutual expression of SERPINE1 and TIMP1 and so might be part of the explanation (see Discussion).

From a previous study by Domenici el al.^9^ we evaluated SERPINE1 vs TIMP1 correlations. The subjects contributing to that study were schizophrenia patients and controls. We calculated correlations for 10,000 random subsets of 200 subjects drawn randomly from 267 control subjects and 229 schizophrenia patients. The results (Supplement Figure S11) indicated patients had far stronger SERPINE1 vs TIMP1 correlations than controls.

### Subnetworks common among all three groups

Several pairs of analytes were highly correlated in all three groups (highlighted in orange in Figs. 2, 3, and 4). Among them was the complete graph formed by interleukins 3, 5, 7, and 13. We examined the correlation pattern of these interleukins in the Domenici data^9^ for 267 unaffected subjects. As shown in the Supplement Figure S9, the four interleukins were highly correlated in that cohort as well.

## Discussion

### Immune correlation networks and psychosis risk

Psychosis conversion in high-risk subjects was characterized by marked simplifications of networks of correlated proteins that regulate tissue remodeling; this might be consistent with the hypothesis of BBB dysregulation in schizophrenia^30^. Notable were graph changes involving SERPINE1. The serine protease inhibitor SERPINE1 is the major inhibitor of the plasminogen activator PLAT; PLAT activates plasminogen to plasmin (the final substrate of the fibrinolytic system) which in turn degrades fibrin. Thus, a focus *infra* is SERPINE1 and related proteins^31^.

Relevant to the present study, schizophrenia patients are subject to increased risk of cardiovascular disease, reduced risk of certain types of cancer, and possibly some aspects of accelerated aging; all these general observations have in turn been associated with SERPINE1 or related proteins^32–36^.

More particularly, connection of schizophrenia with proteins important in coagulation homeostasis has been amplified by recent studies of Hoirisch-Clapauch, Nardi, and their colleagues^37^, starting with anecdotal descriptions of possible benefits to five schizophrenia patients of treatment with warfarin (which would seemingly counter the effects of increased SERPINE1 and decreased PLAT activity). They proposed that normalization of levels of PLAT in the psychotic brain might enable long-term remission of psychotic symptoms.

PLAT is secreted by many cell types including blood vessel endothelial cells that are known to express diverse genes differentially even among regions of the same artery^38–40^. In neurons PLAT occurs in dendrites and synapses; it is stored in pre-synaptic vesicles that cross the synaptic cleft after depolarization, subject to regulation by astrocytes^41,42^.

Aside from hemostasis, PLAT has related roles in smooth muscle signaling in blood vessel tunica^31,43^, several types of ECM signaling^44^, and BBB permeability in the context of cerebral ischemia^45^. Given the large numbers of schizophrenia patients who are comorbid for cardiovascular diseases, who require treatment with warfarin or functionally related pharmaceuticals, and who are not in remission, the intersection of hemostasis and psychosis cannot be simple. However, Hoirisch-Clapauch et al.^34,46^. provided additional reasoning and evidence of a connection, finding lower PLAT levels among 70 schizophrenia patients compared with 98 age-matched controls. Since the principal regulator of PLAT levels is SERPINE1, Hoirisch-Clapauch and Nardi^46^ postulated that inflammatory conditions could increase the risk of schizophrenia through mechanisms involving SERPINE1 levels.

PLAT is highly pleiotropic as, therefore, might be some of its regulators. Two of many reported PLAT substrates directly relevant to psychiatry have functions in epidermal growth factor receptor (EGFR)-mediated neuroprotection^47,48^ and the dynamics of the glutamate receptor and ion channel protein NMDAR (antagonized by phencyclidine in a model of schizophrenia)^49,50^. Furthermore, PLAT is expressed in brain endothelial cells, neurons, and microglia. It is strongly and negatively correlated with mRNA for OCLN (occludin)^51^, a protein important in the stability and permeability of tight junctions. Reactive oxygen species can cause rapid release of PLAT in monocyte and endothelia cocultures^52^. In a related effect (noting the above correlations in unaffected subjects), MMP9 promotes monocyte migration through brain parenchymal basement membrane^52^.

Consistent with our findings, there is also an emerging literature implicating the plasminogen pathway in schizophrenia. This literature points towards down-regulation of PLAT, up-regulation of factors including SERPINE1 that inhibit PLAT activation^34,37,46,53^, and consequences of elevated fibrinogen (fibrin) including incorrect differentiation of progenitors of oligodendrocytes into astrocytes in MS^20^. This misguided differentiation might relate to reported low levels of oligodendrocytes in layer five of prefrontal cortex in schizophrenia^54^.

For data from the same subjects presently studied we previously reported that proteins involved in the plasminogen pathway, including MMP7 and Factor 7, predicted psychosis conversion^3^. In our previous publication we did not find that expression levels of SERPINE1 or TIMP1 were substantially increased going from nonconverters to converters. By contrast, the current study suggests that the extreme changes in correlation networks of SERPINE1 and related proteins, especially TIMP1, may indeed be informative.

Most notable is an interpretation of CTGF in idiopathic pulmonary fibrosis^55,56^ and other diseases^57–59^ that might explain the remarkable correlation of SERPINE1 and TIMP1 levels in converters. Unfortunately, in our blood plasma assays CTGF was not detected in 50–70% of the subjects in all three of the groups, precluding its correlation analysis. However, regarding CTGF as a known driver of SERPINE1 and TIMP1 levels, we note that local upregulation of CTGF by a type of alveolar cell, resulting in proliferation of local fibroblasts, has been proposed as a surrogate biomarker of idiopathic pulmonary fibrosis (IPF);^55^ CTGF is therefore the target of ongoing IPF clinical trials of the fully human monoclonal antibody Pamrevlumab by FibroGen (San Francisco CA) (trial NCT01890265 at ClinicalTrials.gov).

The serine protease inhibitor SERPINE1 is a “suicide protein” in the serpin superfamily and is the major inhibitor of the plasminogen activator PLAT and related protein PLAU (plasminogen activator urokinase). Thus, SERPINE1 is a crucial down regulator of fibrinolysis and ECM degradation^31^. In wound healing, SERPINE1 contributes to regulation of cell proliferation and tissue remodeling^43^, as well as other aspects of cell signaling and migration^44,45^.

Studies of samples from clinical high-risk psychiatric patients in the literature^34^ and in our data (Supplement Figure S10) can exhibit somewhat elevated levels of SERPINE1. Interestingly, de Fouw et al.^60^ reported that the glycoprotein Protein S (PROS1) accelerated neutralization of SERPINE1, thereby increasing PLAT activity; this finding suggests possibilities for indirect control of PLAT levels and function. In its free form, PROS1 is a cofactor of protein C (PROC) in the anticoagulation pathway. PROS1 and PROC are vitamin K-dependent plasma proteins^61^, included among the cofactors in the inactivation of the prothrombinase complex. PROS1 also exists in a complex with complement C4B-binding protein^62^. Complement inhibitor C4B-binding protein enhances plasminogen activation^63^. Isotypes of the C4 protein have been implicated in schizophrenia gene association studies^64^.

SERPINE1 levels are up-regulated by inflammatory cytokines including IL6, TNF, and TGFB1, as reviewed by Brown et al.^65^. These researchers postulated that inflammatory conditions increased the risk of schizophrenia through a combination of mechanisms involving increased SERPINE1 levels or decreased free PROS1 levels.

PLAT was not measured in our assays, but more should be said about some of its extensive functions^66,67^. PLAT degrades ECM and in particular cleaves the large glycoprotein RELN, all contributing to cell migration and tissue remodeling, particularly revascularization. PLAT affects neural, endothelial, and glial cells, driving multiple, sometimes opposing effects by activating or otherwise regulating diverse transcription factors and receptors. Regarding the neurotrophin BDNF (brain-derived neurotrophic factor), PLAT promotes the cleavage of neuronal proBDNF to mature BDNF, a function essential for late-phase long-term potentiation^68^. Pro- and mature BDNF have important and opposing effects on synaptic plasticity, regulation of neurogenesis, and neuronal survival^69^. Only the mature BDNF was assayed by us; it is prominent in Figs. 2–4.

MMP9 is also an important factor in ECM maintenance and remodeling, assayed herein both as mature MMP9 and as the total of proMMP9 and mature MMP9 (labeled MMP9t in our graphs). It regulates glutamate receptors, modulates physiological and morphological synaptic plasticity, and is regulated by glutamate at excitatory synapses^70^. MMP9 and other extracellular proteases such as plasmin convert proBDNF to mature BDNF^71^. PLAT, PLAU, plasmin, and MMP9 all have roles in BBB disruption after stroke^72^. MMP9 itself is activated in presence of oxidative stress and in turn promotes the receptor for advanced glycation end-product (RAGE) (to be described in a forthcoming paper by D. Dwir et al.), said to induce a self-reinforcing cycle of inflammatory responses and further oxidative stress^73,74^. In addition, MMP9 is involved in the degradation of the perineuronal net, a type of ECM that wraps fast-spiking parvalbumin interneurons; the perineuronal net is known to be affected in the medial prefrontal brain of schizophrenia patients^75,76^. In mice, intravenously administered PLAT was detected within the brain parenchyma and cerebrospinal fluid, having crossed the BBB by transcytosis^77^.

In summary, for converter subjects SERPINE1 was no longer correlated with antithetical MMPs, but instead became highly correlated with the synthetical TIMP1. This observation and the above integration of these proteins and CTGF in multiple disorders suggest the potential importance of CTGF inhibition. Several inhibitors of CTGF have been proposed and studied; for example, insulin and an siRNA have been studied in the context of inhibition of vascular remodeling in diabetic retinopathy^78^. However, certain advantages attend a monoclonal antibody for CTGF inhibition.

### Interleukin networks common in all subjects

We found that IL3, IL5, IL7, and IL13 form a highly correlated module in all groups, and replicated this finding in an external test set of unaffected subjects (Figure S8), suggesting co-regulation and closely related functions of these cytokines. Three genes, *IL3*, *IL5*, and *IL13* (and also *IL4*), exist as a cytokine gene cluster on chromosome arm 5q. Unfortunately, IL4 detection was weak (below LLOQ) or missing in more than half our samples, precluding correlation analysis with the rest of that cluster. But IL4 and IL7 are among the six “γ-chain utilizing” interleukins, while IL3, IL4, IL5, and IL13 are the four “IL4-like” interleukins; both sets of proteins are considered tightly packed α-helices in a four-helix bundle motif of short core helices^79^. There are about 45 human interleukins, so the members of the identified correlation cluster are more similar than would be expected by chance.

The top shared pathway for IL3, IL5, IL7, and IL13 from application of IPA was “hematopoiesis from multipotent stem cells” (*p* = 1.6E-9). IL3 promotes hematopoietic stem cell proliferation, and IL7 regulates development of the common lymphoid progenitor cells and development of B-cell, T-cell, and NK-cell lineages. In detail, IL3 regulates development of the common myeloid progenitor, and IL5 regulates development of neutrophils and eosinophils^80^. Less is known regarding IL13, but its receptor, IL12A1, is selectively expressed on CD62L+ cells (common lymphoid progenitors), again suggesting a role in regulation of lymphoid cell differentiation^81^. Normally, the circulating proportions of neutrophils, lymphocytes, monocytes, and eosinophils are relatively constant, so possibly IL3, IL5, IL7, and IL13 in our graphs contribute to maintenance of homeostatic proportions of those cell types.

## Limitations

Foremost among limitations, the sample sizes of the three groups in our study were all small. We plan to add data from more subjects in the near future (NAPLS3). In addition, the Luminex platform is less reproducible for certain proteins than other platforms, including Meso-Scale, ELISA, and microfluidic ELISA. We plan to employ microfluidic ELISA in pending analyses. Another limitation is the two-year limit for the definition of conversion. Although the rate of psychosis conversion is much lower after two years, we anticipate that up to 10% of our nonconverters will eventually convert to psychosis, implying some uncertainty in all our statements about nonconverters vs converters. However, by considering only networks constructed from extremely high correlations and thousands of random subsets of the groups, we consider it unlikely that the findings could be explained entirely by chance alignments. Lastly, many proteins functionally related to those in our assays were not considered due to costs. Therefore, important signals might be missing from our analyses. Despite all the seemingly plausible relationships described *supra*, our findings need confirmation and expansion in other cohorts. Further work of multiple types, e.g., as that being undertaken by Sorokin^82^, is required to directly link peripheral immune dysregulation to the etiology of schizophrenia.

## Translation to the clinic

The present work suggests that additional longitudinal studies of clinical high-risk patients with logically expanded assays of agents of immune responses could lead to parsimonious lists of proteins implicated in conversion to schizophrenia. Ratios of distinguishing proteins for a new patient could be compared with historical ratios to predict membership among nonconverters or converters.

Furthermore, the above potential connection of SERPINE1 and TIMP1 expression with CTGF regulation might lead to investigation of Pamrevlumab (http://www.fibrogen.com/pamrevlumab-trials/) or related agents of CTGF inhibition. The potential medical value of inhibition of CTGF in various diseases has long been recognized. The recent appearance of Pamrevlumab as a potential treatment for IPF and its repurposing suggested herein for prevention of conversion to psychosis might be especially important. Repurposing is a drug development strategy with huge savings of time and expense.

## Electronic supplementary material

Supplemental Material
