# Supplementary material for: Networks of blood proteins in the neuroimmunology of schizophrenia
Source: Transl Psychiatry. 2018 Jun 6;8:112. doi: 10.1038/s41398-018-0158-y (PMC5990539; doi:10.1038/s41398-018-0158-y)
Supplement: Supplementary file 1 — Supplemental Material [file 41398_2018_158_MOESM1_ESM.docx]

Supplemental Material

1. Figure S1. Schematic trajectories in NAPLS data. Page 2

2. Figure S2. Flowcharts for data from the three groups. Page 3

3. Figure S3. Comparison of strong correlations among analytes in unaffected comparison and nonconverter groups. Page 4

4. Figure S4. Comparison of strong correlations among analytes in nonconverter and converter groups. Page 5

5. Figure S5. Edges representing correlations in unaffected comparison subjects that at least 500 times in 10000 trials exceeded a strong threshold. Page 6

6. Figure S6. Edges representing correlations in nonconverter subjects that at least 500 times in 10000 trials exceeded a strong threshold. Page 7

7. Figure S7. Edges representing correlations in converter subjects that at least 500 times in 10000 trials exceeded a strong threshold. Page 8

8. Figure S8. Consistency as graph edge counts of strong correlations among analytes.

Page 9

9. Figure S9. Comparison of interleukin correlations from Domenici et al.^1^ vs the present study for unaffected comparison subjects. Page 10

10. Figure S10. Histograms of SERPINE1 vs TIMP1 correlation values. Page 11

11. Figure S11. SERPINE1 vs TIMP1 correlations from Domenici data.^1^ Page 12

12. Terms of SIPS, SOPS, and COPS scale systems. Page 13

13. References Page 14


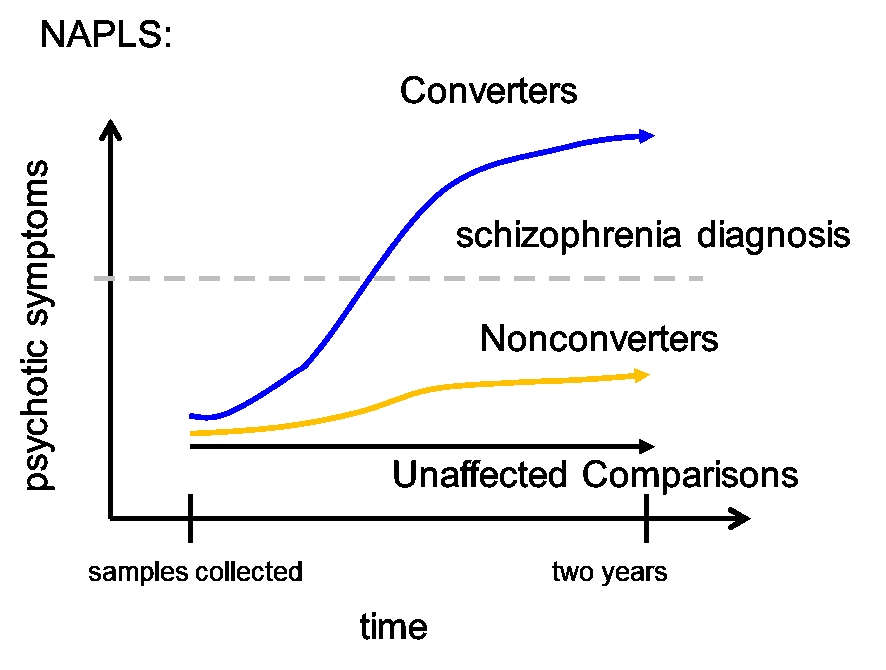


Figure S1. Representation of NAPLS data. Shown schematically are typical trajectories for clinical high-risk patients who converted to schizophrenia or a related psychotic disorder within two years, other patients who did not convert, and unaffected comparison subjects. In the present study, patients' blood plasma samples were collected during prodrome states. Thus, the present longitudinal study differs from many other psychiatric biomarker reports that compare samples from patients with a diagnosis of schizophrenia vs unaffected comparison subjects.


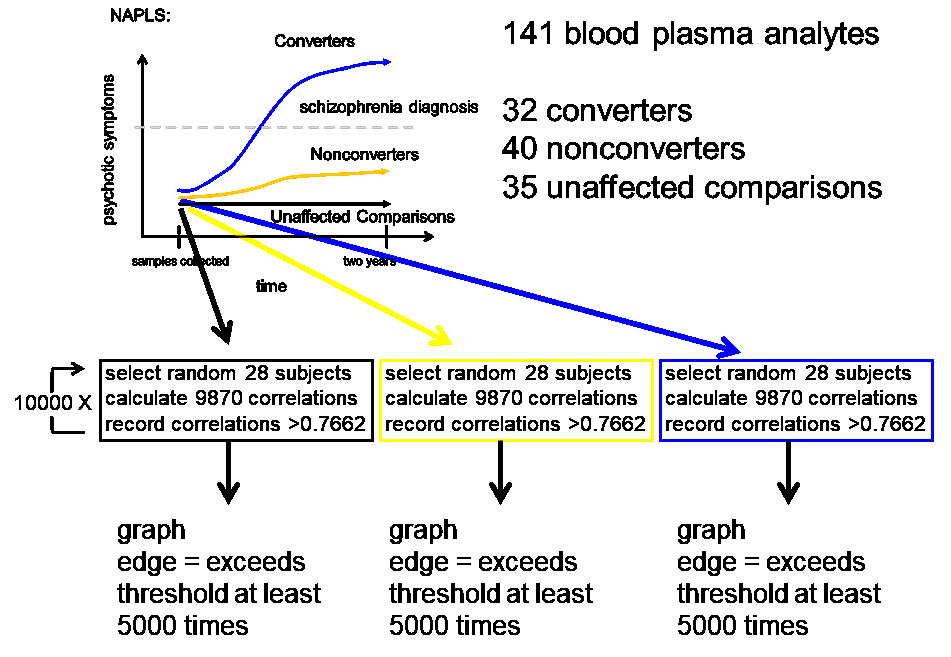


Figure S2. Flowcharts for data from the three groups leading to graphs.


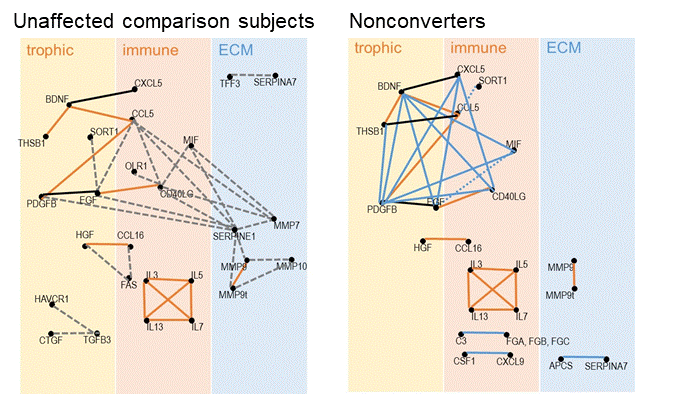


Figure S3. Comparison of networks between unaffected comparison subjects and nonconverters. Proteins are organized by their main function whenever possible. Edges represent robust correlations that are preserved in the three groups (orange), lost going from unaffected comparisons to nonconverters (dashed), or gained going from unaffected comparisons to nonconverters (blue). Note in nonconverters the loss of correlations involving analytes related to ECM and the gain of correlations involving trophic factors.


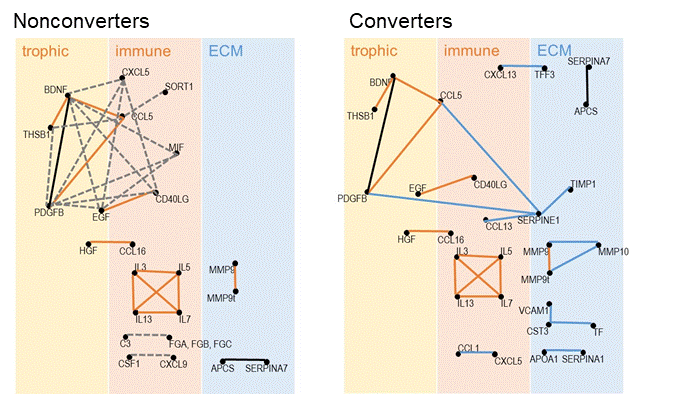


Figure S4. Comparison of networks between nonconverters and converters. Proteins are organized by their main function whenever possible. Edges represent robust correlations that are preserved in the three groups (orange), lost going from nonconverters to converters (dashed) or gained going from nonconverters to converters (blue). Note in converters the loss of correlations involving trophic factors and the gain of correlations involving proteins related to ECM (proteins that are mostly different from those in unaffected graph).


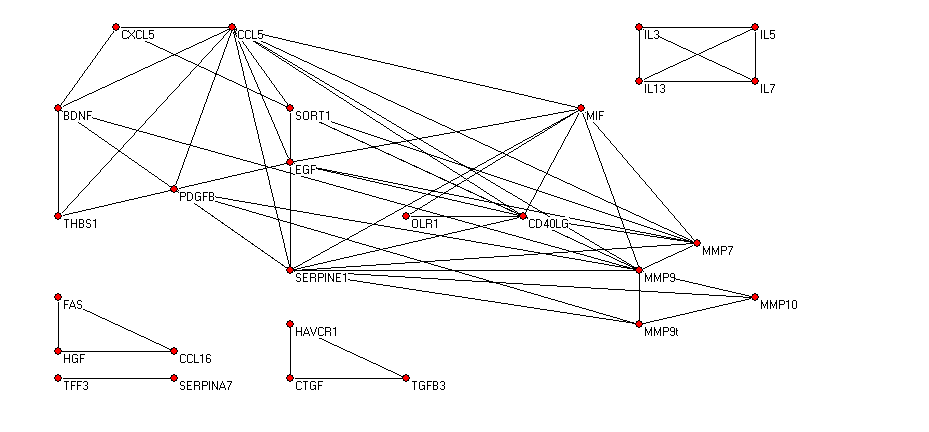


Figure S5. Edges for unaffected comparison subjects that represent correlations that in at least 500 of 10000 random draws of 28 subjects had a values exceeding 0.7662. Blood plasma proteins are labeled by their gene common symbols. Shown are 27 vertices and 60 edges, yielding an edge to vertex ratio = 2.22 and a normalized edge complexity = 0.171. SERPINE1 is in 10 edges while TIMP1 is absent.


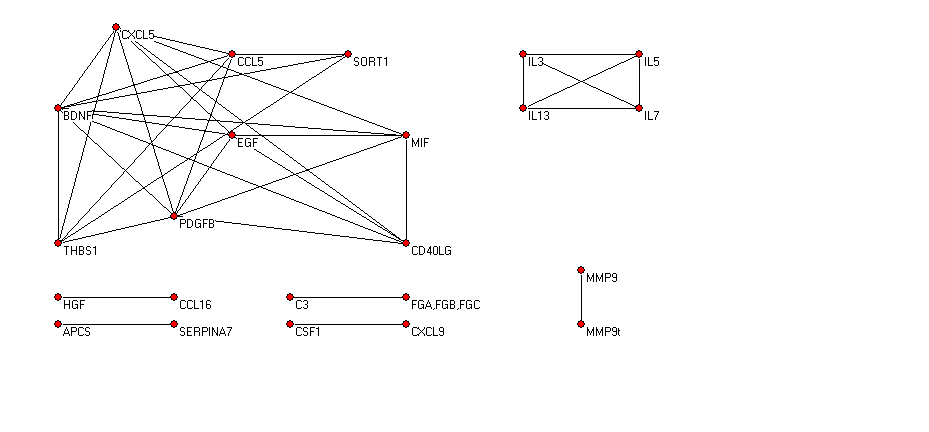


Figure S6. Edges for nonconverters that represent correlations that in at least 500 of 10000 random draws of 28 subjects had a values exceeding 0.7662. Shown are 23 vertices and 36 edges, yielding an edge to vertex ratio = 1.57 and a normalized edge complexity = 0.142. SERPINE1 and TIMP1 are both absent.


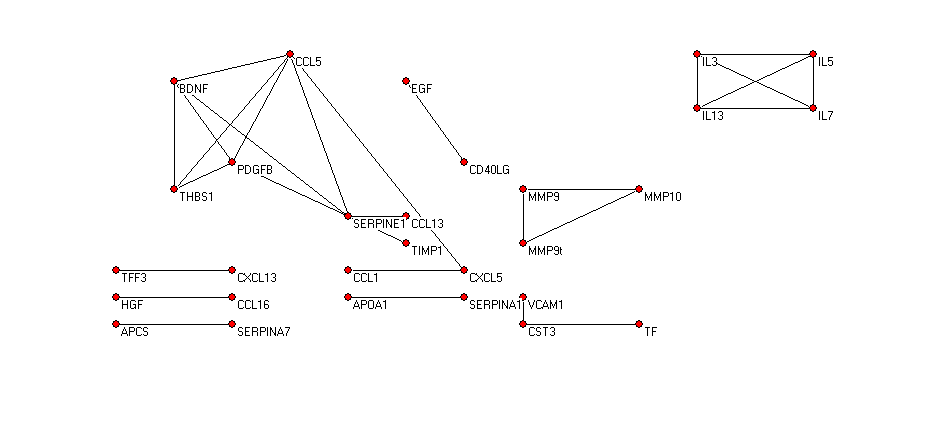


Figure S7 Edges for converters that represent correlations that in at least 500 of 10000 random draws of 28 subjects had a values exceeding 0.7662. Shown are 29 vertices and 29 edges, yielding an edge to vertex ratio = 1.00 and a normalized edge complexity = 0.071. SERPINE1 is the vertex in the greatest number of edges (5). Unlike the other two groups, SERPINE1 is strongly correlated with TIMP1, the only edge including TIMP1. As noted in the text, the SERPINE1 to TIMP1 correlations exceed the threshold > 8400 times in 10000 draws (hence certainly > 500), a very consistent signal.

Dysregulation of inflammatory pathways may play a role in nonconverters as well as in converters. Inflammation can be linked to redox dysfunction and oxidative stress.

Notably, though Figures S5 and S6 have multiple MIF edges, Figure S7 has none. Beside its pro-inflammatory action, MIF also plays an important role as redox regulator. Dysregulation of MIF can impact growth factors such as BDNF, PDGF, and EGF, and by regulating cell growth and regeneration, MIF may modulate disease conversion.

In summary, graphs S5, S6, S7 suggest that in using a more lenient threshold for edge inclusion (500 of 10000 instead of 5000 of 10000, as in Figures 2, 3, 4), additional unaffected comparison edges constitute the main difference.


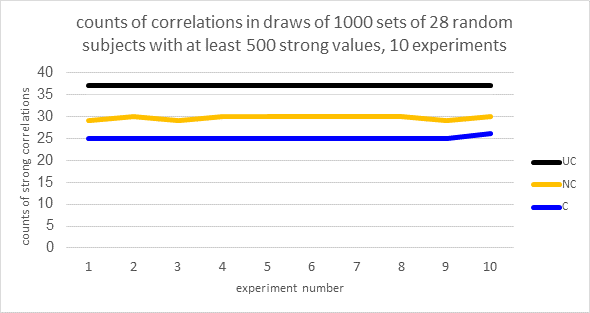


Figure S8. Consistency as graph edge counts of strong correlations among analytes. We analyzed a total of 1000 random selections of 28 subjects ten times from each of the three groups. As defined for this graph, a strong correlation of a pair of distinct analytes (among 9870 pairs from 141 analytes) meant that, for at least 500 selections of 1000, the Pearson correlation was > 0.7662 as in the preparation of graphs in Figures 2, 3, 4.


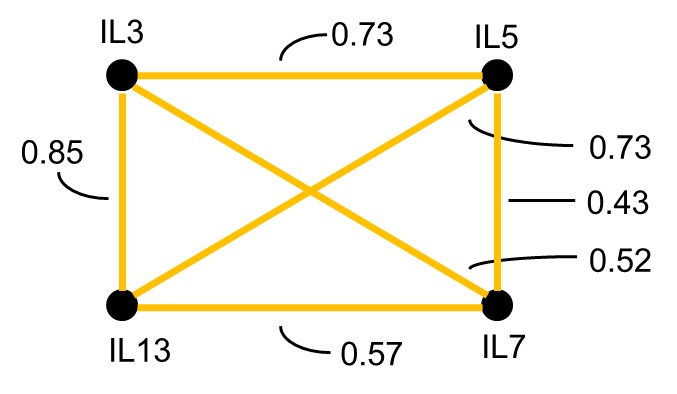


Figure S9. Strong correlations from 267 Unaffected comparison subjects and 58 markers in an external test set of data from Domenici et al.^1^. Among the four interleukins mutually correlated in Figures 2, 3, 4 in the main text, the same six strong correlations also appearing in the Domenici data. The shown correlations are much higher than can be explained by chance in 267-dimensional space.


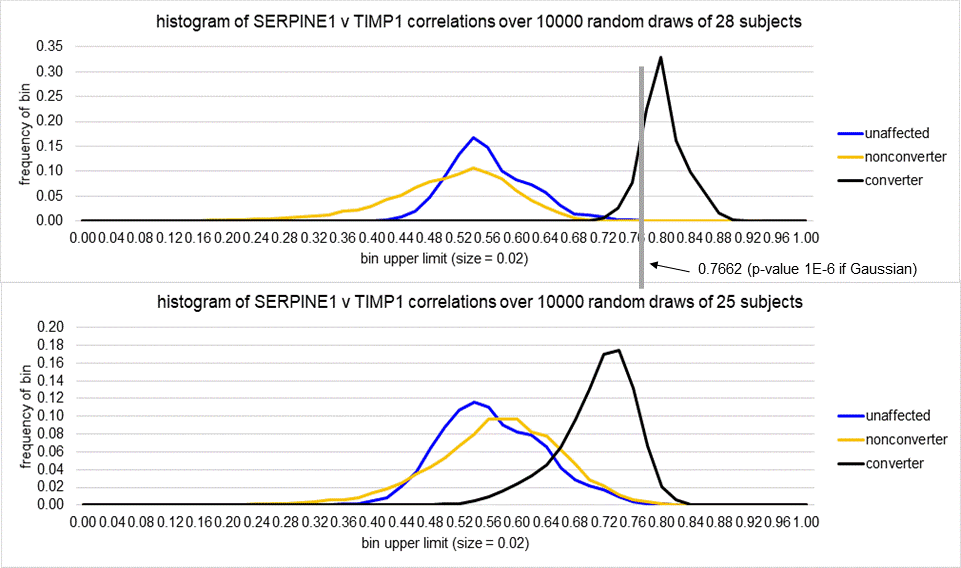


Figure S10. SERPINE1 vs TIMP1 correlations. Above is a histogram of 10000 trials of correlations over random 28-subject subsets of the three groups. Eighty-four percent of the correlations from converter subjects exceed the threshold 0.7662 (gray bar). However, few or none of the correlations from unaffected comparison or nonconverter subjects do the same. Below is a graph of the same information with random draws of only 25-subject subsets. Since fewer subjects are used (a loss of information in correlation calculations), there is a loss of discrimination, but the three groups are still distinguished in the same way.


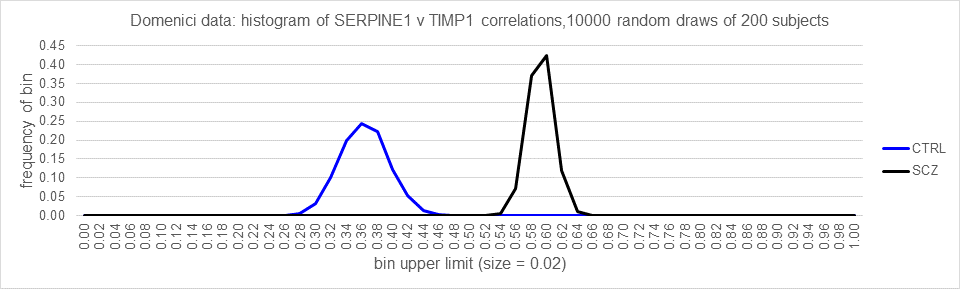


Figure S11. SERPINE1 vs TIMP1 correlations from Domenici data^1^. The data are not from a longitudinal study but are comparisons of unaffected comparison subjects or controls (CTRL) vs patients with schizophrenia (SCZ). A total of 10000 times subsets of 200 subjects were randomly drawn from 267 controls or 229 patients. As in Figure S10, the histogram again shows dramatically higher correlations for schizophrenia.

Terms of structured interviews and scales

Structured interviews and scales are discussed by Miller et al.^2^.

The Structured Interview for Prodromal Symptoms (SIPS) contains the Scale of Prodromal Symptoms (SOPS) that rates the severity of relevant symptoms with the following scale: 0 = absent, 1 = questionably present, 2 = mild, 3 = moderate, 4 = moderately severe, 5 = severe but not psychotic, and 6 = severe and psychotic. The SOPS is composed of four symptom domains that are classified as: positive (unusual thought content, suspiciousness, grandiose ideation, perceptual abnormalities, disorganized communication); negative (social anhedonia, avolition, expression of emotion, experience of emotions and self, ideational richness, occupational functioning); disorganized (odd behavior or appearance, bizarre thinking, trouble with focus and attention, impairment in personal hygiene); and general (sleep disturbance, dysphoric mood, motor disturbances, impaired tolerance to normal stress).

Criteria of Prodromal States (COPS) includes ratings of three clinical syndromes.

(1) Attenuated Positive Symptom: new onset or recent worsening of subsyndromal (“attenuated”) positive psychotic symptoms,

(2) Brief Intermittent Psychosis: very brief periods (lasting minutes) of fully psychotic positive symptoms, or

(3) Genetic Risk with Deterioration: deterioration in functioning within the last year and schizotypal personality disorder (SPD) or a having first-degree relative with psychosis.

Typically, over 90% of subjects meet the Attenuated Positive Symptom syndrome.

References

1 Domenici, E. *et al.* Plasma protein biomarkers for depression and schizophrenia by multi analyte profiling of case-control collections. *PLoS One* **5**, e9166, doi:10.1371/journal.pone.0009166 (2010).

2 Miller, T. J. *et al.* Prodromal assessment with the structured interview for prodromal syndromes and the scale of prodromal symptoms: predictive validity, interrater reliability, and training to reliability. *Schizophr Bull* **29**, 703-715 (2003).
